# Case Report: Palliative benefit of hypofractionated radiation therapy (6 Gy × 6 fractions) with temporary tracheostomy in a cat with laryngeal B-cell lymphoma progressing during chemotherapy

**DOI:** 10.3389/fvets.2026.1855062

**Published:** 2026-09-10

**Authors:** Jingyu Kim, Jaewon Kim, Inseong Jeong, Hoon Choi, Younghwan Kim, Suhyung Lee, Kidong Eom, Jaehwan Kim

**Affiliations:** 1 Department of Veterinary Medical Imaging, College of Veterinary Medicine, Konkuk University, Seoul, Republic of Korea; 2 Royal Animal Medical Center, Seoul, Republic of Korea; 3 Department of Veterinary Pathology, College of Veterinary Medicine, Konkuk University, Seoul, Republic of Korea

**Keywords:** cat, hypofractionated radiation therapy, laryngeal lymphoma, radiotherapy, temporary tracheostomy

## Abstract

A 12-year-old spayed female Maine Coon cat was diagnosed with cervical T-cell-rich B-cell lymphoma. During modified L-CHOP-based chemotherapy, the cat developed progressive upper airway obstruction associated with a laryngeal lesion. Initial CT suggested contiguous laryngeal involvement, and follow-up imaging revealed severe laryngeal narrowing. An emergency temporary tracheostomy was performed for airway stabilization, and subsequent punch biopsy of the laryngeal lesion was most consistent with diffuse large B-cell lymphoma. Hypofractionated palliative radiation therapy (6 Gy × 6 fractions; total dose 36 Gy) was initiated to address the obstructive mass. Progressive tumor shrinkage was observed during treatment, and by the fourth fraction the tracheostomy tube was successfully removed without recurrence of airway obstruction. Follow-up CT 17 days after radiation therapy initiation showed no visible laryngeal lesion, and no respiratory signs or airway obstruction recurred before the cat died on day 27 following clinically suspected systemic disease progression. This case suggests that hypofractionated radiation therapy, in combination with temporary tracheostomy, may provide rapid and effective relief of tumor-induced upper airway obstruction in cats with laryngeal B-cell lymphoma progressing during chemotherapy.

## Introduction

Feline lymphoma is the most commonly diagnosed malignant tumor in cats, accounting for approximately one-third of all feline cancers (1–3). Extranodal forms now represent the predominant presentation, and the anatomical site of involvement strongly influences clinical behavior, therapeutic approach, and prognosis (1). Within this spectrum, laryngeal lymphoma is an uncommon manifestation but may present with acute, life-threatening upper airway obstruction (1, 2).

Typical clinical signs include increased respiratory effort, upper airway noise such as stridor or stertor, and voice change or loss, often accompanied by non-specific signs such as lethargy and reduced appetite (1, 2). Diagnostic imaging is useful for assessing lesion extent and airway compromise, whereas definitive diagnosis requires cytologic or histopathologic confirmation. However, laryngeal lymphoma can be difficult to distinguish from inflammatory or infectious laryngitis on imaging alone, as both may present as soft tissue thickening of the laryngeal region (4).

In cases of severe upper airway obstruction, temporary tracheostomy may be required to bypass the obstruction and stabilize the patient prior to definitive treatment (5–7). Although tracheostomy in cats is associated with a relatively high complication rate, particularly due to tube obstruction and dislodgement, recent evidence suggests that successful management is achievable in most cases, with mortality more commonly related to the underlying disease rather than the procedure itself (5–7). In contrast, permanent tracheostomy, which is intended for long-term airway diversion, has been associated with a guarded prognosis and challenges in long-term management in cats (5, 6).

Treatment options for feline laryngeal tumors include chemotherapy, surgery, and radiation therapy, used alone or in combination depending on tumor characteristics (8). Lymphoma is generally considered both chemosensitive and radiosensitive, and localized upper respiratory tract lymphoma often responds favorably to multi-agent chemotherapy and/or radiation therapy (1, 2, 9–12). However, when local disease persists or progresses during chemotherapy, achieving adequate local control can be challenging, and radiation therapy may serve as an important adjunct or alternative modality.

Definitive radiation therapy (DRT) has been reported to achieve favorable local control in human patients with laryngeal lymphoma (13, 14). Although evidence in veterinary medicine remains limited, definitive-intent radiation therapy has also been associated with favorable local control in feline laryngeal tumors (15), and stereotactic radiation therapy has been successfully applied in cats with laryngeal neoplasia (16).

In addition, hypofractionated palliative radiation therapy is widely used to achieve rapid clinical improvement and alleviate obstructive signs, particularly in patients with advanced or progressive disease (17). However, to the authors’ knowledge, there are no previous reports describing the combined use of temporary tracheostomy and palliative external beam radiation therapy for feline laryngeal lymphoma presenting with acute upper airway obstruction.

Therefore, this case report describes a cat with laryngeal B-cell lymphoma progressing during chemotherapy, in which temporary tracheostomy combined with hypofractionated palliative radiation therapy provided rapid relief of life-threatening respiratory distress. This case highlights the potential role of combined airway stabilization and local radiotherapy as a palliative strategy in similar clinical scenarios.

### Case description

A 12-year-old, spayed female Maine Coon cat presented with lethargy, anorexia, and abnormal respiratory sounds. On physical examination, the cat was bright, alert, and responsive, with a body weight of 5.78 kg and a body condition score of 3/9. A firm, non-movable mass was palpated in the right cervical region, with bilateral mandibular lymphadenopathy. Other findings were unremarkable.

**Figure 1:**
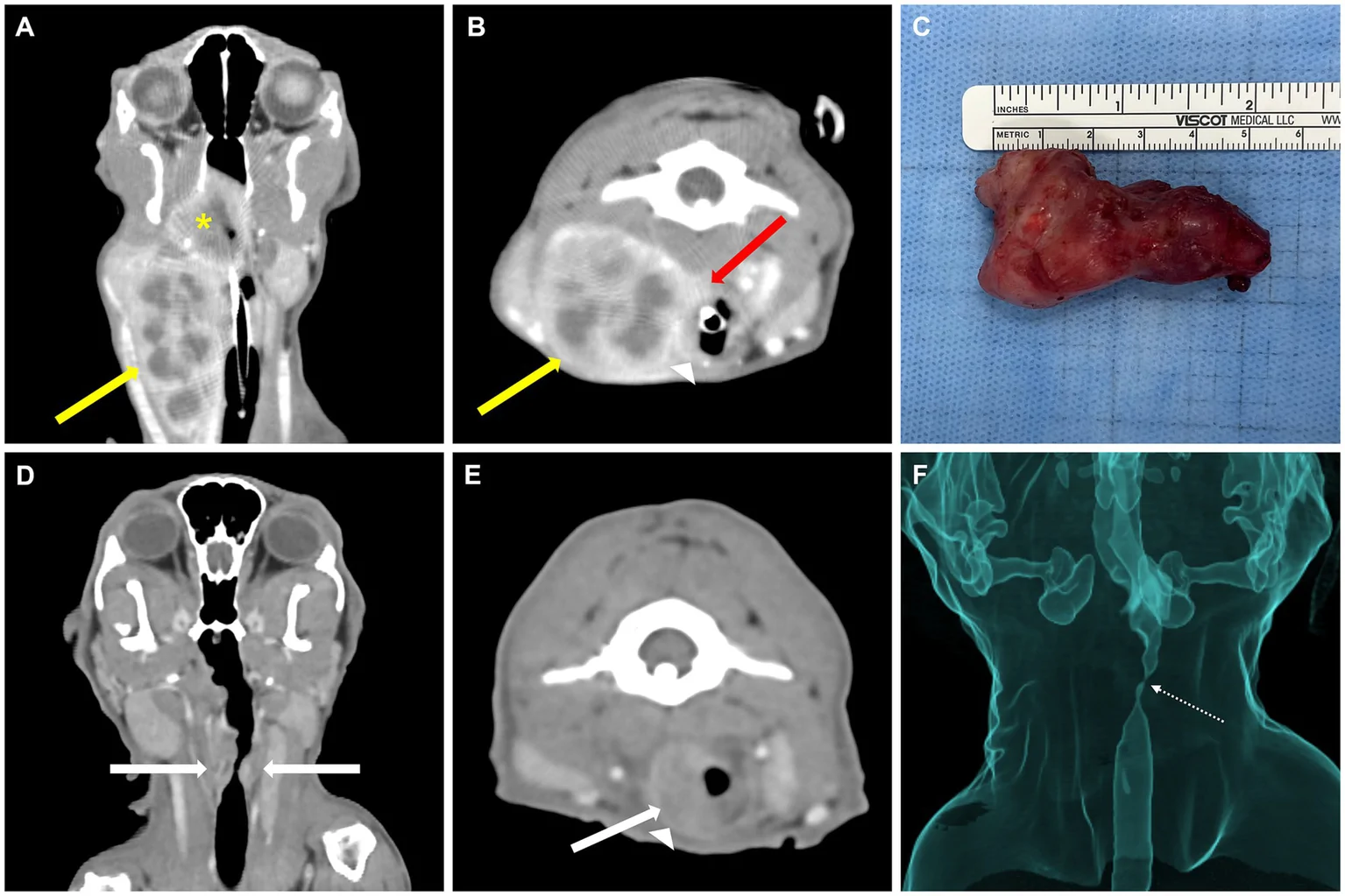
Initial distribution of the cervical and tonsillar lesions and subsequent progression of the laryngeal lesion. **(A)** Dorsal multiplanar reconstruction CT image from the initial examination showing the right tonsillar mass (yellow asterisk) and right cervical mass (yellow arrow). **(B)** Transverse CT image from the initial examination showing the right cervical mass (yellow arrow) and suspected contiguous extension into the laryngeal soft tissues (red arrow). The thyroid cartilage is indicated by a white arrowhead. **(C)** Gross photograph of the surgically debulked cervical component of the mass. **(D)** Dorsal multiplanar reconstruction CT image obtained without anesthesia after worsening of the respiratory signs during modified L-CHOP-based chemotherapy, showing diffuse inward thickening of the laryngeal soft tissues (white arrows) and marked narrowing of the airway lumen. **(E)** Corresponding transverse CT image showing the laryngeal soft-tissue thickening (white arrow) and airway narrowing. The thyroid cartilage is indicated by a white arrowhead. **(F)** Three-dimensional CT reconstruction of the airway from the examination performed without anesthesia, demonstrating an abrupt and marked reduction in airway diameter at the level of the larynx (dotted white arrow).

Hematologic and biochemical analyses at initial presentation revealed mild hyperkalemia, hyperglycemia, and increased feline serum amyloid A (fSAA) concentrations. Initial pre- and post-contrast computed tomography (CT) images were acquired using a 16-slice helical CT scanner (Activion 16; Toshiba Medical Systems Corporation, Otawara, Japan) with the following parameters: 120 kVp, 135 mAs, and a slice thickness of 1.0 mm. The examination revealed two distinct soft tissue masses: one in the right tonsillar region and the other in the right cervical region (Figures 1A,B). The right cervical mass extended medially toward the laryngeal soft tissues, raising suspicion of contiguous laryngeal involvement (Figure 1B). The cervical component of the mass was surgically debulked (Figure 1C), and tissue samples were collected for histopathological and immunohistochemical examination. These analyses identified a round cell neoplasm most consistent with T-cell-rich B-cell lymphoma. In contrast, the right tonsillar lesion was diagnosed as severe stomatitis.

Based on these findings, a modified L-CHOP-based chemotherapy protocol was initiated. The cat completed one cycle of the protocol from day 0 to day 21, consisting of L-asparaginase (400 IU/kg, SC) on day 0; vincristine (0.7 mg/m^2^, IV) on days 1 and 15; cyclophosphamide (250 mg/m^2^, IV) on day 8; and epirubicin (25 mg/m^2^, IV) on day 21, with epirubicin administered in place of doxorubicin. Prednisolone was initiated on day 1 at 2 mg/kg PO q24h and tapered to 1 mg/kg PO q24h from day 15 onward. Mild improvement in respiratory signs was observed following surgical debulking and initiation of chemotherapy; however, the improvement was transient, and the respiratory signs subsequently recurred and progressively worsened during the treatment cycle.

Because of the progressive respiratory distress, further evaluation was performed using fluoroscopy, laryngoscopy, and CT. Pre- and post-contrast CT images were acquired without anesthesia using a 160-slice multidetector CT scanner (Aquilion Lightning; Canon Medical Systems Corporation, Otawara, Japan) with the following parameters: 120 kVp, 135 mAs, and a slice thickness of 1.0 mm. The CT examination demonstrated diffuse inward thickening of the laryngeal soft tissues, resulting in marked narrowing of the upper airway lumen (Figures 1D–F). Concurrently, multiple enlarged cervical lymph nodes and multifocal round, hypoattenuating hepatic nodules were identified, raising concern for systemic lymphomatous involvement.

Due to severe airway compromise, an emergency temporary tracheostomy was performed to stabilize the patient. Three days later, a planning CT scan and punch biopsy of the laryngeal mass were performed. The cat was positioned in dorsal recumbency and immobilized using a customized vacuum cushion (Chunsung, Seoul, Republic of Korea). Pre- and post-contrast CT images were acquired using a 64-slice helical CT scanner (Optima CT660; GE HealthCare, Chicago, IL, USA) with the following parameters: 120 kVp, 160 mA, and a slice thickness of 1.25 mm. Intravenous contrast medium (iohexol, Omnipaque 300; GE HealthCare) was administered at a dose of 600 mg iodine/kg via a power injector (MEDRAD Stellant; Bayer, Leverkusen, Germany). Histopathological findings of the laryngeal mass, together with immunohistochemical staining for CD3 and CD20, were most consistent with diffuse large B-cell lymphoma.

Based on these findings, hypofractionated palliative radiation therapy was selected for local tumor control. Inverse treatment planning was performed using Eclipse software (version 13.7.33; Varian Medical Systems). A total dose of 36 Gy was prescribed in six fractions of 6 Gy, delivered twice weekly. The gross tumor volume (GTV) was defined as the contrast-enhancing portion of the laryngeal mass and enlarged regional lymph nodes. The clinical target volume (CTV) was generated by applying a 2-mm isotropic expansion to the GTV to account for microscopic disease extension. The planning target volume (PTV) was created by applying an additional 2-mm isotropic expansion to the CTV to account for setup uncertainties and organ motion.

**Figure 2:**
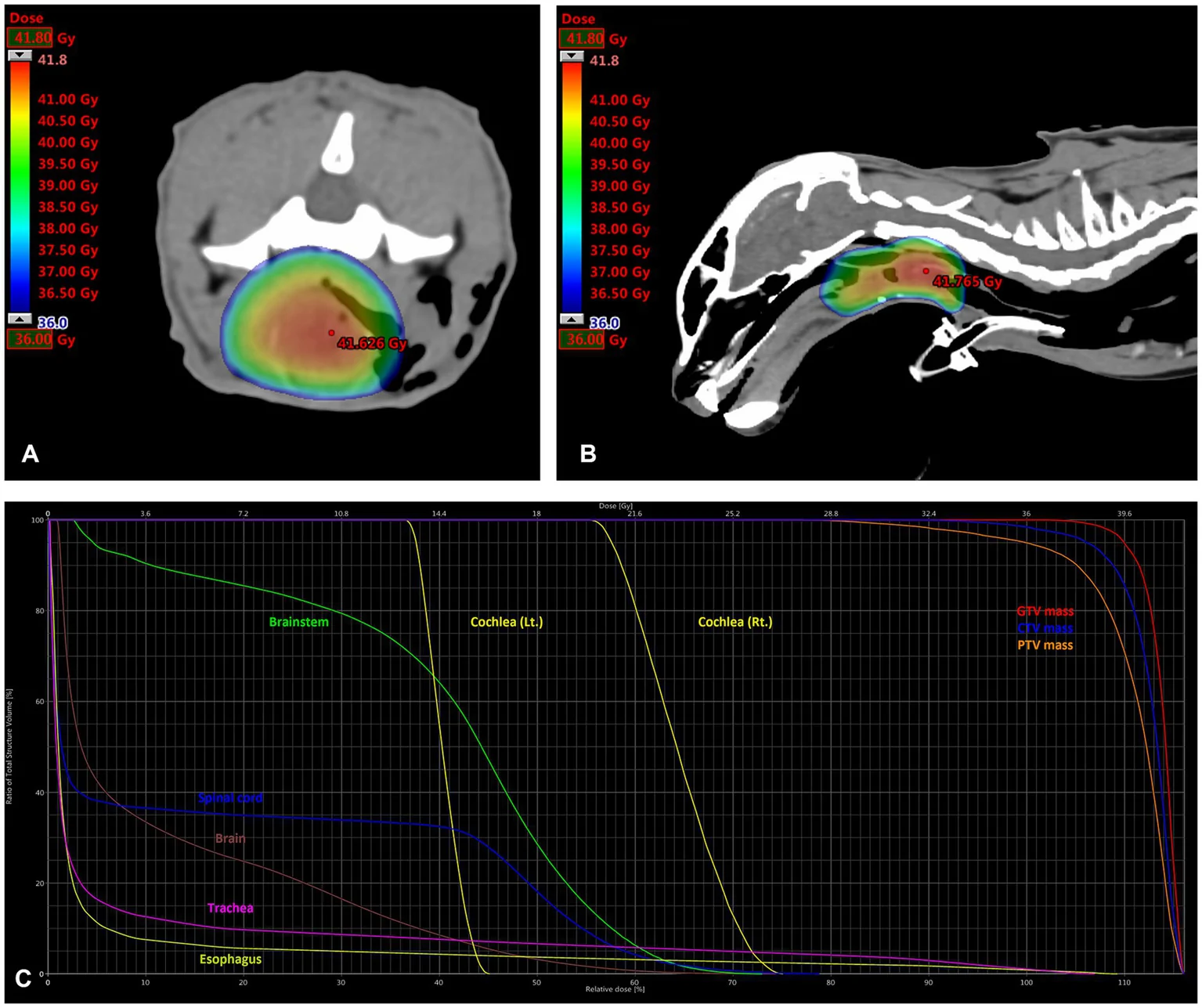
Radiation therapy planning performed using Eclipse™ software (version 13.7.33). **(A)** Transverse and **(B)** sagittal CT images showing dose distribution within the laryngeal mass. The color wash represents the radiation dose distribution, with warmer colors indicating higher doses. **(C)** Dose–volume histogram (DVH) illustrating target coverage and dose distribution to organs at risk (OARs), including the trachea, esophagus, brain, spinal cord, brainstem, and cochlea. The treatment plan ensured adequate dose coverage of the gross tumor volume (GTV) and planning target volume (PTV) while minimizing exposure to surrounding normal tissues.

Organs at risk (OARs), including the trachea, esophagus, brain, brainstem, spinal cord, and cochlea, were contoured, and portions of the PTV overlapping these structures were adjusted to minimize radiation exposure. For the laryngeal mass, treatment planning ensured that 100% of the GTV mass and at least 95% of the PTV mass received at least 95% of the prescribed dose (Figure 2), as summarized in Table 1.

**Table 1:** Summary of dose and volume data.

Structure	Volume [cm^3^]	Dmax[Gy]	Dmean[Gy]	Dmin[Gy]	D2%[Gy]	D50%[Gy]	D98%[Gy]
GTV mass	5.8	41.8	40.9	36.3	41.7	41.1	38.9
CTV mass	12.0	41.8	40.4	31.8	41.6	40.8	36.3
PTV mass	19.6	41.8	39.8	27.5	41.6	40.4	32.6
GTV LN	0.7	41.1	39.3	30.9	41.0	39.9	34.5
CTV LN	2.1	41.2	38.1	22.2	41.1	39.5	28.0
PTV LN	4.5	41.2	36.6	17.1	41.1	39.1	21.1
PTV Total	23.5	41.8	39.1	17.1	41.6	40.2	27.9
Esophagus	6.4	39.3	1.9	0.05	30.4	0.3	0.07
Trachea	6.5	38.5	2.9	0.06	34.1	0.3	0.07
Brain	30.6	26.7	4.3	0.3	19.3	1.2	0.36
Brainstem	3.1	26.2	14.5	0.9	23.1	15.9	1.1
Spinal cord	6.7	28.3	6.6	0.03	23.2	0.5	0.03

GTV, gross tumor volume; CTV, clinical target volume; PTV, planning target volume; LN, lymph node; Dmax, maximum dose; Dmean, mean dose; Dmin, minimum dose; D2%, dose received by 2% of the volume; D50%, dose received by 50% of the volume; D98%, dose received by 98% of the volume. All doses are reported in Gray (Gy).

Two days after the planning CT scan, hypofractionated palliative radiation therapy was initiated using volumetric modulated arc therapy (VMAT) and delivered with a 6 MV linear accelerator equipped with 5 mm multileaf collimators (Clinac iX; Varian Medical Systems, Palo Alto, CA, USA).

### Outcomes

By the fourth fraction, cone-beam CT acquired for treatment verification demonstrated a marked reduction in the laryngeal mass with restoration of the airway lumen, allowing removal of the temporary tracheostomy tube without recurrence of upper airway obstruction.

**Figure 3:**
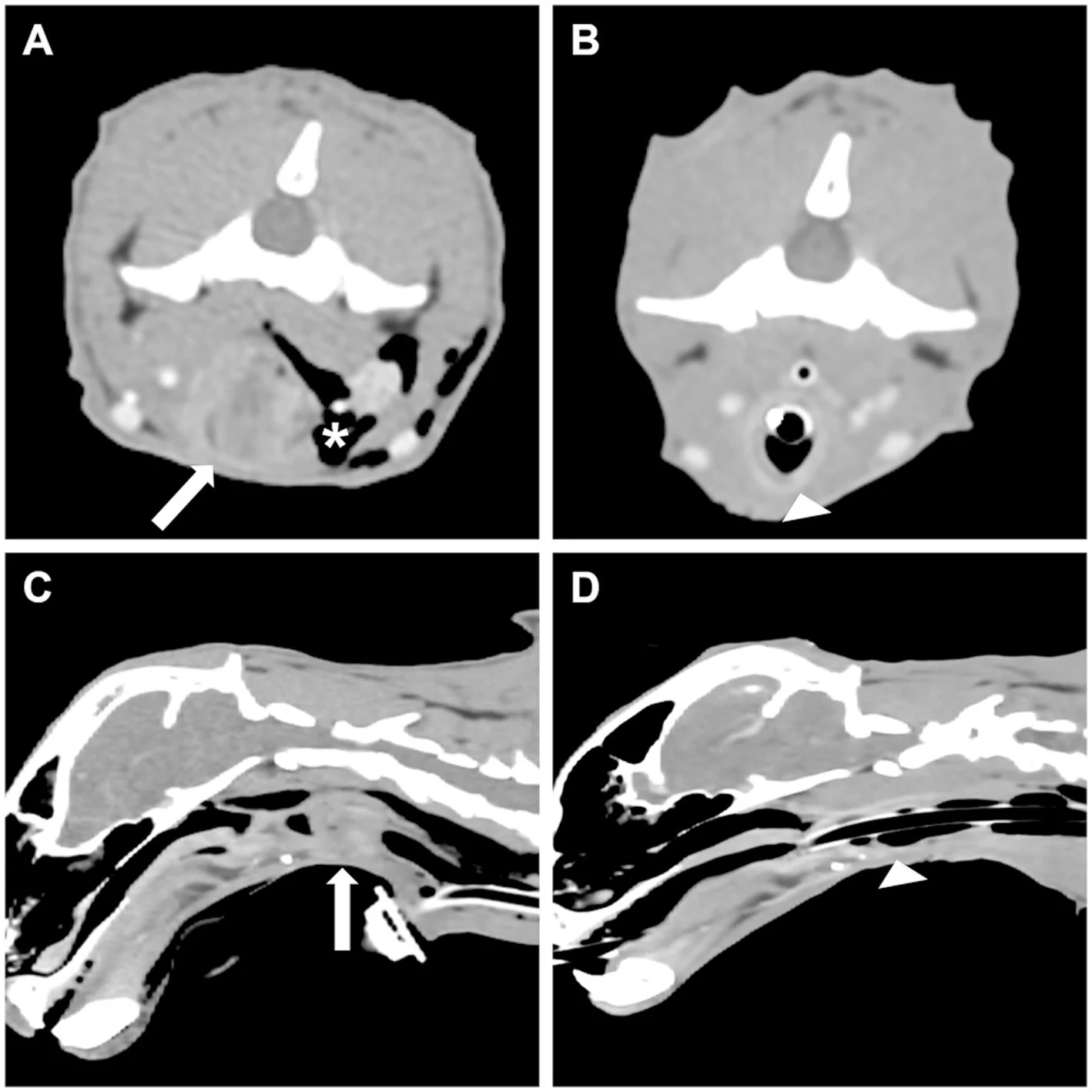
Computed tomography (CT) images demonstrating treatment response of the laryngeal mass **(A,C)** Planning CT images obtained prior to radiation therapy **(B,D)** Follow-up CT images obtained 17 days after initiation of radiation therapy, following completion of treatment. **(A)** Transverse planning CT image showing a laryngeal mass resulting in complete loss of the airway lumen (white arrow). Subcutaneous emphysema associated with the tracheostomy procedure is indicated by an asterisk (*). **(B)** Transverse CT image obtained after completion of radiation therapy showing no discrete residual laryngeal mass and restoration of the airway lumen (arrowhead). **(C)** Sagittal planning CT image demonstrating the laryngeal mass (white arrow). **(D)** Sagittal CT image obtained after completion of radiation therapy showing no discrete residual laryngeal mass with preservation of normal laryngeal anatomy (arrowhead).

Follow-up CT (Optima CT660; GE HealthCare, Chicago, IL, USA), performed 17 days after initiation of radiation therapy following completion of treatment, demonstrated a marked reduction in the laryngeal lesion. The pretreatment GTV for the laryngeal mass measured 5.8 cm^3^, whereas the soft-tissue volume within the corresponding laryngeal region measured approximately 0.8 cm^3^ on follow-up CT. No discrete residual laryngeal mass was identified, and only normal-appearing laryngeal soft-tissue structures remained (Figure 3). These findings supported a marked local response, with sustained relief of upper airway obstruction throughout the remaining survival period.

Despite the marked locoregional response, the same CT examination revealed findings suspicious for systemic disease progression, including an increase in the size and number of multifocal hypoattenuating hepatic nodules and the appearance of new bilateral hypoattenuating renal nodules, all considered suspicious for lymphomatous involvement.

The cat received a single dose of lomustine (10 mg/cat, PO) and was subsequently discharged. However, the cat died 27 days after initiation of radiotherapy following clinically suspected systemic disease progression rather than respiratory deterioration. Notably, no recurrence of upper airway obstruction or acute radiation-related toxicity was observed between completion of radiotherapy and death.

## Discussion

This case describes a cat with laryngeal B-cell lymphoma progressing during chemotherapy, presenting with life-threatening upper airway obstruction, in which the combined use of temporary tracheostomy and hypofractionated palliative radiation therapy resulted in rapid clinical improvement and clinically meaningful short-term palliation. Restoration of airway patency occurred early during radiation therapy, allowing timely removal of the tracheostomy tube and limiting the duration of exposure to tracheostomy-related complications.

Laryngeal lymphoma is an uncommon manifestation of feline lymphoma but may lead to acute upper airway obstruction requiring emergency intervention (1, 2). Although high response rates to chemotherapy have been reported in cats with extranodal lymphoma, survival outcomes may vary depending on tumor location (1, 3, 12, 18). In the present case, respiratory signs and laryngeal narrowing progressed during multi-agent chemotherapy, highlighting the need for an alternative local palliative treatment.

Radiation therapy is a well-established modality for lymphoma due to its high radiosensitivity, particularly in localized disease (2, 13, 14, 18). Hypofractionated protocols are widely used to achieve rapid symptom relief and improve quality of life (17). In this case, marked tumor reduction was observed by the fourth fraction, resulting in resolution of airway obstruction and enabling removal of the tracheostomy tube. This supports the potential role of hypofractionated radiation therapy in providing urgent palliation for obstructive laryngeal lymphoma progressing during chemotherapy.

Palliative radiation therapy protocols are inherently variable, with multiple fractionation schedules reported, including once- or twice-weekly regimens as well as alternative approaches such as quad shot protocols (17). Treatment selection is often individualized based on clinician preference and patient-specific factors (17). Although a once-weekly schedule is commonly used for 6 Gy × 6 protocols, a twice-weekly regimen was selected in the present case to facilitate more rapid tumor reduction in the setting of life-threatening airway obstruction. This accelerated schedule was considered clinically justified given the need for urgent airway relief, and notably, rapid tumor regression was achieved without evidence of acute radiation-related toxicity.

Despite the marked local response, the cat died in the setting of suspected systemic disease progression. This outcome highlights a limitation of local therapy for feline extranodal lymphoma: although radiation therapy may achieve effective control of the irradiated lesion, it does not address occult or emerging disease outside the treatment field. Therefore, comprehensive staging and consideration of systemic therapy remain important when local radiation therapy is selected (1). A similar clinical pattern has been reported in a cat with primary intratracheal lymphoma that developed systemic lymphoma despite sustained control of respiratory signs following local treatment (11).

In this cat, temporary tracheostomy served as a short-term bridge to radiation therapy by stabilizing the life-threatening upper airway obstruction until sufficient tumor reduction restored airway patency. While the tracheostomy tube was in place, two episodes of acute respiratory distress occurred because of tube obstruction by fibrous material, each requiring urgent intervention. This was consistent with tube obstruction being an important complication of temporary tracheostomy in cats (5, 6). By the fourth radiation fraction, cone-beam CT demonstrated marked reduction of the laryngeal mass and restoration of the airway lumen, allowing removal of the tracheostomy tube. No recurrence of upper airway obstruction occurred after tube removal. This clinical course highlights the value of temporary tracheostomy as a bridging intervention while also supporting removal as soon as airway patency has been sufficiently restored.

The cervical lesion was classified as T-cell-rich B-cell lymphoma, which is characterized by neoplastic B cells within a prominent background of reactive T lymphocytes (19), whereas the laryngeal lesion was classified as diffuse large B-cell lymphoma. Because their biological relationship could not be established without clonality or molecular analysis, the discussion focused on the clinical course and local treatment response of the laryngeal lesion.

Several limitations should be considered. Rapid tumor volume reduction during the course of radiation therapy, together with subcutaneous emphysema associated with the tracheostomy procedure and the transition to orotracheal intubation following tracheostomy closure, introduced potential uncertainties in the positioning of both the gross tumor volume (GTV) and adjacent organs at risk (OARs) relative to the initial planning CT.

To mitigate these factors, image-guided radiation therapy (IGRT) was employed to verify target positioning and ensure adequate coverage of the GTV within the planning target volume (PTV) at each treatment session. Although the clinical impact of these uncertainties was likely limited given the palliative intent and marked tumor regression, the use of adaptive radiation therapy to account for anatomical and volumetric changes may have further improved treatment accuracy and dose delivery. Additionally, this is a single case report, and long-term follow-up was not available to confirm the durability of local tumor control.

In conclusion, hypofractionated palliative radiation therapy produced a rapid and marked local response, restored airway patency, and provided clinically meaningful short-term palliation of life-threatening airway obstruction in this case. Temporary tracheostomy served as a short-term stabilization bridge until radiation-induced tumor reduction permitted tube removal, thereby limiting the duration of exposure to tube-related complications. This combined approach may represent a practical and clinically valuable palliative strategy, particularly in emergency settings requiring rapid airway stabilization in cats with obstructive laryngeal lymphoma progressing during chemotherapy.

## Data Availability

The original contributions presented in the study are included in the article/supplementary material, further inquiries can be directed to the corresponding author.

## References

[1] Moore A. Extranodal lymphoma in the cat: prognostic factors and treatment options. J Feline Med Surg. (2013) 15:379–90. doi: 10.1177/1098612X13483236, 23603501 PMC10816586

[2] Rodriguez-Piza I Borrego JF Treggiari E Verganti S Priestnall SL Lara-Garcia A. Clinical presentation, treatment and outcome in 23 cats with laryngeal or tracheal lymphoma. J Feline Med Surg. (2023) 25:1098612X221143769. doi: 10.1177/1098612X221143769, 36655881 PMC10812051

[3] Teske E Van Straten G Van Noort R Rutteman GR. Chemotherapy with cyclophosphamide, vincristine, and prednisolone (COP) in cats with malignant lymphoma: new results with an old protocol. J Vet Intern Med. (2002) 16:179–86. doi: 10.1111/j.1939-1676.2002.tb02352.x, 11899035

[4] Kurihara M Yoshida S Suematsu M. Diagnostic imaging features of inflammatory laryngeal disease in cats. Vet Radiol Ultrasound. (2025) 66:e70014. doi: 10.1111/vru.70014, 39963991

[5] Guenther-Yenke CL Rozanski EA. Tracheostomy in cats: 23 cases (1998-2006). J Feline Med Surg. (2007) 9:451–7. doi: 10.1016/j.jfms.2007.06.002, 17693112 PMC10911508

[6] Elmenhorst K Villedieu EJA Cantatore M Rossanese M Baines SJ. Complications and outcomes of temporary tracheostomy in 24 cats. J Small Anim Pract. (2026) 67:541–7. doi: 10.1111/jsap.70093, 41589552

[7] Moser J Haimel G Tichy A Findji L. Partial laryngectomy for the management of laryngeal masses in six cats. J Feline Med Surg. (2022) 24:373–80. doi: 10.1177/1098612X211027488, 34236002 PMC10812240

[8] North SM Banks TA. "Tumours of the larynx and trachea, mediastinum, chest wall and cardiopulmonary system". In Eds. Susan M. North and Tania Ann Banks. Small Animal Oncology. Edinburgh: Elsevier (2009). p. 115–6.

[9] Kim S Hwang G Kim JY Yun CO Lee S Choi M . Successful treatment of feline nasopharyngeal lymphoma by hypofractionated radiation therapy after surgical debulking in a cat. J Vet Clin. (2024) 41:117–22. doi: 10.17555/jvc.2024.41.2.117

[10] Sfiligoi G Théon AP Kent MS. Response of nineteen cats with nasal lymphoma to radiation therapy and chemotherapy. Vet Radiol Ultrasound. (2007) 48:388–93. doi: 10.1111/j.1740-8261.2007.00262.x, 17691642

[11] Brown MR Rogers KS Mansell KJ Barton C. Primary intratracheal lymphosarcoma in four cats. J Am Anim Hosp Assoc. (2003) 39:468–72. doi: 10.5326/0390468, 14518655

[12] Taylor SS Goodfellow MR Browne WJ Walding B Murphy S Tzannes S . Feline extranodal lymphoma: response to chemotherapy and survival in 110 cats. J Small Anim Pract. (2009) 50:584–92. doi: 10.1111/j.1748-5827.2009.00813.x, 19891724

[13] Coşkun Ç Kurucu N Kutluk T Yılmaz T Bulut E Üner A. Primary B-cell non-Hodgkin lymphoma of the larynx in children: report of two cases and a review of the literature. Turk J Pediatr. (2025) 67:765–71. doi: 10.24953/turkjpediatr.2025.6042, 41327991

[14] Chraa FZ Ezzouitina C Laraichi R Nouni K Lachgar A El Kacemi H . A rare case report of primary laryngeal diffuse large B-cell lymphoma. Eur J Med Health Sci. (2025) 7:42–5. doi: 10.24018/ejmed.2025.7.4.2360

[15] Sesanto C Lawrence J Holkham J Serra JC Bavcar S Parys M. Long-term outcome following multimodality treatment in a cat with recurrent laryngeal adenocarcinoma. Vet Radiol Ultrasound. (2025) 66:e13466. doi: 10.1111/vru.13466, 39681987 PMC11649849

[16] Fujiwara-Igarashi A Goto S Nakazawa Y Ohshima T Tetsu H Miura K . Long-term remission in a cat with laryngeal squamous cell carcinoma treated with stereotactic radiation therapy. J Feline Med Surg Open Rep. (2026) 12:1–6. doi: 10.1177/20551169261419878, 41797734 PMC12966511

[17] Tollett MA Duda L Brown DC Krick EL. Palliative radiation therapy for solid tumors in dogs: 103 cases (2007–2011). J Am Vet Med Assoc. (2016) 248:72–82. doi: 10.2460/javma.248.1.72, 26684094

[18] Meier VS Beatrice L Turek M Poirier VJ Cancedda S Stiborova K . Outcome and failure patterns of localized sinonasal lymphoma in cats treated with first-line single-modality radiation therapy: a retrospective study. Vet Comp Oncol. (2019) 17:528–36. doi: 10.1111/vco.12517, 31254440

[19] Day MJ Kyaw-Tanner M Silkstone MA Lucke VM Robinson WF. T-cell-rich B-cell lymphoma in the cat. J Comp Pathol. (1999) 120:155–67. doi: 10.1053/jcpa.1998.0267, 10087489

